# Global protein profiling of human milk using pre-enriched RNA-sequence libraries

**DOI:** 10.1038/s41598-026-41374-w

**Published:** 2026-03-03

**Authors:** Julie Astono, Asger Givskov Jørgensen, Claus Bus, Søren Drud-Heydary Nielsen, Jørgen Kjems, Ulrik Kræmer Sundekilde

**Affiliations:** 1 Department of Food Science, Aarhus University, Agro Food Park 48, Aarhus N, Denmark; 2 Department of Molecular Biology and Genetics, Interdisciplinary Nanoscience Center, Aarhus University, Gustav Wieds Vej 14, Aarhus C, Denmark; 3 Arla Food Ingredients, Sønderupvej 26, Videbæk, Denmark

**Keywords:** Biochemistry, Biological techniques, Biomarkers, Molecular biology

## Abstract

The complex protein composition of human milk remains challenging to characterize due to technical limitations, yet such insights are crucial as early-life nutrition influences long-term health outcomes and is associated with pathological conditions such as obesity. In this study, we applied APTASHAPE, a high-throughput profiling method based on chemically modified protein-binding RNA molecules, to globally profile protein composition in skimmed human milk and to explore associations with maternal and infant characteristics. A total of 520 human milk samples collected at 3 days, 1 month, 2 months, and 3 months postpartum were analyzed. Ordinary least squares regression analysis of the discovery cohort identified discriminatory RNA sequence profiles reflecting the global protein composition and correlating with sampling time, maternal BMI, and parity. These associations were subsequently validated and confirmed in an independent test cohort. Furthermore, 14 candidate aptamers were subjected to protein pull-down assays followed by mass spectrometry, which identified C4b-binding protein and tenascin C as candidate targets associated with maternal BMI and sampling time. Overall, our findings highlight the dynamic nature of the human milk protein composition, with the greatest variation attributable to sampling time, while maternal BMI and parity accounted for more subtle differences.

**Supplementary Information:**

The online version contains supplementary material available at 10.1038/s41598-026-41374-w.

## Introduction

Human milk proteins are essential for infant development, supplying vital amino acids for growth, supporting the maturation of both the innate and adaptive immune systems, and facilitating the establishment of a healthy initial gut microbiota^1^. Many studies have investigated total protein content and its variability related to sampling time, maternal and infant characteristics, including maternal pre-pregnancy body mass index (BMI), maternal age, gestational age, infant sex, and infant birth weight^2–6^. It is well established that the total protein content of human milk declines over the course of lactation, primarily due to the evolving physiological needs of the infant, from immunological support in the early postnatal period to increased energy and macronutrient demands for growth, coinciding with rising concentrations of lactose and lipids^7^.

The composition of the milk and early life nutrition is also explored in the context of obesity, which is a worldwide crisis leading to obesity-related diseases and thereby causing enormous societal economic costs^8,9^. Studies have shown that infants fed with formula milk have a higher risk of becoming obese later in life^10^. The higher risk of developing obesity has been related to the higher amount of protein in formula compared to human milk^11^. Furthermore, the risk of childhood obesity becomes higher if the mother is overweight^12^. However, the link and possible intergenerational transmission (genetic, epigenetic, or environmental) between mother and child has not been conclusively established. Direct investigations focusing on the relationship between maternal obesity and human milk proteins yielded inconsistent results^2,3,13^. Most studies focus on the total protein concentration or specific proteins such as lactoferrin^3,14^. Regarding total protein, both negative and positive correlations to maternal BMI have been observed^2,3^, along with studies not finding a correlation^4^. One study found 210 significantly different proteins when comparing human milk from overweight and obese women^15^, while another study used matrix-assisted laser desorption ionization time-of-flight (MALDI-TOF) and found decreased diversity in the human milk protein profile of overweight/obese compared to normal weights^16^. While these studies provide valuable insights, comprehensive profiling of the human milk proteome has been performed to a much lesser extent, largely due to the complexity of the human milk matrix and technical challenges.

**Fig. 1:**
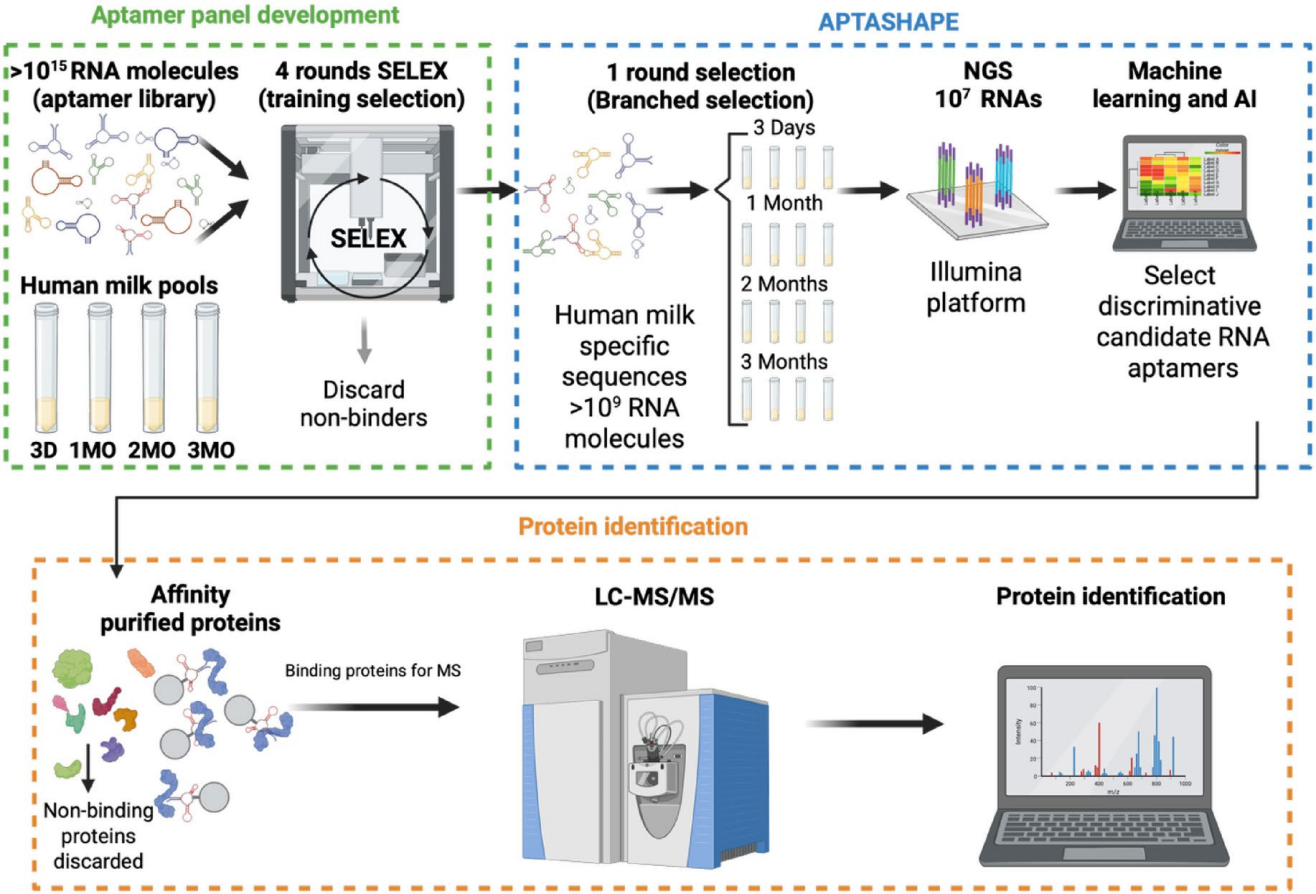
Schematic representation of the experimental workflow. Candidate *aptamer panel development*: A fully random RNA library is presented to mixtures of human milk from samples capturing variation at the sampling time and is enriched for human milk protein binding RNA molecules through 4 rounds of SELEX, where the non-binding RNA molecules are discarded. *APTASHAPE*: The four human milk protein-specific RNA libraries (3 days, 1 month, 2 months, 3 months) obtained after the third SELEX round are mixed equimolar, and the mixed library is presented to individual human milk samples in parallel (branched selection). Analysis of protein-binding RNA sequences using next-generation sequencing yields quantitative data reflecting the global protein profile in each human milk sample and identifies condition-specific RNA sequence profiles. The APTASHAPE step is performed on the discovery cohort to identify discriminatory RNA sequence profiles, and is then repeated on an independent validation cohort to confirm their discriminatory capacity. *Protein identification*: Selected discriminatory RNA sequences (candidate aptamers) are immobilized on magnetic beads to capture RNA sequence-specific proteins from human milk samples. The resulting pull-down fractions are then analyzed by mass spectrometry, and proteins are identified by searching against curated libraries of human milk proteins and peptides. *NGS* next generation sequencing, *SELEX* systematic evolution of ligands by exponential enrichment.

In this study, we apply APTASHAPE, a high-throughput protein profiling platform that leverages chemically modified, 2′-fluoro-modified, RNA (RiboNucleic Acid) molecules to interact with proteins in complex biofluids (RNA aptamer library)^17^. APTASHAPE enables large-scale, quantitative profiling of the global protein composition, offering a scalable and sensitive approach that also accounts for single amino acids substitution and post-translational modifications^18^. Until now, APTASHAPE has successfully discriminated between disease states and studied biomarkers of bladder cancer, COVID-19, and metabolic dysfunction-associated steatotic liver disease^17,19,20^. The overall workflow is outlined in Fig. 1. The APTASHAPE experiments were performed on a discovery cohort (n = 344) and a validation cohort (n = 176). The 520 total samples represent different collection times, from 3 days, 1, 2, and 3 months postpartum, and were obtained from the Danish cohort Maternal Infant Health (MaInHealth), which was established to investigate the natural variation in human milk and the possible effect on the infant^21^. The RNA molecules with affinity toward the proteins (candidate aptamers) in the samples are partitioned and analyzed using next-generation sequencing (NGS), leading to sample-specific RNA sequence binding profiles and ultimately identification of condition-specific RNA sequence profiles^17–20^.

## Materials and methods

### Participants and sample collection

Participants providing human milk samples for the study are a part of the Danish cohort MaInHealth, and recruitment took place between May 2019 and December 2021. The cohort is registered at ClinicalTrials.gov (identification number: NCT05111990 and https://clinicaltrials.gov/study/NCT05111990), with a registration date of November 8, 2021 (First Posted), and approved by the Central Denmark Regional Committees of Health Research Ethics (approval reference: 1-10-72-296-18). All participants provided informed consent in accordance with the Declaration of Helsinki II. All exclusion and inclusion criteria have been described earlier^21^. Characteristics related to the birth were obtained from the electronic health journal, and all maternal and infant characteristics provided after 1 month postpartum were self-reported via electronic questionnaires^21^. In short, mothers should intend to breastfeed for the first 4–6 months, be healthy and not suffer from any chronic diseases, not have a planned cesarean section (c-section), and give birth at term, defined as after 37 weeks of gestation. After birth, formula use was allowed up to four times a week, further details are provided elsewhere^21^.

Human milk samples were collected at 3 days, 1, 2, and 3 months postpartum. Participants were given instructions to manually express human milk in a sterile 40 mL container at least 2 hours (h) after the last feeding, around midday, and to avoid the first few drops. Participants stored the samples in their freezer until pickup within 14 days after collection. The samples were transported on dry ice to the Department of Food Science, Aarhus University, where they were stored at − 70 °C until further analysis. Samples were thawed and centrifuged at 4000×*g* for 10 min at 4 °C, and 100 µL skim milk was transferred to a 96-well plate and stored at − 70 °C.

### SELEX and APTASHAPE protocol

The preparation of random RNA libraries, the enrichment of these libraries toward human milk proteins, i.e., selection of protein-binding RNA molecules (candidate aptamers), cDNA synthesis, polymerase chain reaction (PCR) amplification, regeneration of the RNA library by in vitro transcription, and branched selections were performed as previously described with minor modifications^17^. All SELEX selections were started from aliquots of the same, well-mixed RNA master library. The RNA master library was prepared from a commercial ordered ssDNA template library through Klenow primer extension using an input corresponding to 3.85 × 10^15^ molecules (6.4 nmol ssDNA). Following in vitro transcription and urea-PAGE purification, the RNA master library yield was approximately ~ 4.5 × 10^16^ molecules (74.7 nmol). Therefore, each aliquot reliably represents the same statistical distribution of sequences. The iterative rounds of SELEX were performed on four human milk pools (3 days, 1, 2, and 3 months postpartum), each consisting of 12 samples, to obtain a sequence library able to bind the diverse set of proteins present in human milk. In the positive selection step, 10 µL human milk containing proteins was immobilized on 200 µg *N*-hydroxysuccinimide(NHS)-activated magnetic beads, and binding RNA molecules were partitioned. This was done to guide the RNA library toward human milk-specific characteristics. Additionally, to enhance human milk characteristics and remove matrix binding RNA molecules were, bovine serum albumin (BSA) was immobilized on 200 µg of NHS-activated magnetic beads in the negative selection step. Non-binding RNA molecules from the negative selection were incubated with the positive selection matrix containing human milk proteins. Based on the retained sequence diversity, defined as the number of distinct RNA sequences detected by NGS, the RNA library from the third selection round was selected for branched selections.

For APTASHAPE, the RNA libraries recovered from each sample after branched selections were prepared for NGS sequencing using forward and reverse PCR primers containing sample-specific barcodes, Illumina NGS adaptors sequence, and Illumina NGS primer sequences, allowing for multiplexing. The total cDNA amount of each sample was evaluated using qPCR, and samples were mixed equimolar followed by gel purification and sequenced on the Illumina NovaSeq machine (Novaseq S1, 300 (400–500 Gb, 1.3–1.6 B reads) v1.5**)** according to the manufacturer protocol. The run included 50% Phix control library (Illumina # FC-110-3001). The output from the Illumina sequencing was processed as previously described^17^, generating a table containing the sequences and the sequence count for all RNA sequences in each sample.

### Analysis pipeline

The data was analyzed using the in-house developed analysis pipeline previously described with minor modifications^17^. The *p* values from the ordinary least squares (OLS) analysis were adjusted using the Benjamini–Hochberg method with a false discovery rate of 0.05%, and a 0.2 threshold change in regression coefficient between the investigated conditions was applied. The calculation of the Damerau–Levenshtein distance for the joining of highly similar sequences was done using the stringdist package (v.0.9.15)^22^. The data was visualized in boxplots using the ggpubr package (v0.6.0), and the statistical significance in median PC1 values was determined by Wilcoxon rank-sum testing^23^. Experimental scripts are available in the ERDA repository, https://anon.erda.au.dk/cgi-sid/ls.py?share_id=a3W2AgCRwf.

### Protein pull-down

Human milk proteins were affinity-purified using 3′-biotinylated candidate aptamers immobilized on streptavidin-coated magnetic beads and then incubated with pooled human milk. Candidate aptamers (1 nmol RNA) were biotinylated in 1× reaction buffer (50 mM Tris–HCl pH 7.5, 10 mM MgCl_2_, 10 mM DTT, 1.5 mM ATP, 50% PEG8000), 50 units T4 RNA ligase (Thermo Fischer Scientific™, cat.no. EL0021), 6 µM final pCp-biotin (Jena Bioscience cat.no. NU-1706-BIO), and 0.5 units inorganic pyrophosphatase (Thermo Scientific™ cat.no. EF0221). The reaction was incubated for 16 h at 4 °C. According to manufacturer protocol, biotinylated candidate aptamers were purified using RNA Clean and Concentrator-25 spin columns (Zymo Research cat.no. R1017). Biotinylated RNA (400 pmol) was immobilized on 100 µL streptavidin magnetic beads (Invitrogen™ cat. no. 11205D) in 1× HBS buffer (150 mM NaCl, 20 mM HEPES, pH 7.4) for 30 min. Beads were washed 3 times in 100 µL 1× HBS buffer supplemented with 1% tRNA, followed by incubation in 1× HBS buffer supplemented with 50% human milk for 30 min, and washed 3 times in 100 µL 1 HBS buffer. Bound proteins were eluted by resuspension of pellets in 1× HBS buffer supplemented with 20 mM EDTA and incubated for 10 min at 45 °C, shaking.

### Mass spectrometry

Samples were resolubilized in 40 µL 6 M urea in 50 mM Tris–HCl. Dithiothreitol was added to a final concentration of 30 mM and incubated for 30 min at room temperature. Subsequently, iodoacetamide was added to a final concentration of 15 mM and incubated in dark for 30 min at room temperature. For digestion, a trypsin/Lys-C mix was added at a 25:1 protein:protease ratio (w/w), mixed and incubated for 3 h at 37 °C. Urea concentration was diluted to less than 1 M and incubation was continued overnight. The digestion was terminated by adding trifluoroacetic acid (TFA) to a final concentration of 1%. Particulate material was removed by centrifugation at 14,000×*g* for 10 min. Samples were dried using vacuum centrifugation.

The peptides were cleaned and desalted using Pierce™ peptide desalting spin colums (Thermo scientific) according to the manufacturer’s instructions. Briefly, the columns were conditioned using acetonitrile and washed using 0.1% TFA. Samples were resuspended in 0.1% TFA, loaded on the column, centrifuged at 3000×*g* for 1 min, and washed 3 times with 0.1% TFA. Peptides were eluted over two rounds using 50% acetonitrile, 0.1% TFA. Samples were evaporated to dryness using vacuum centrifugation. 

Peptides were resuspended in 2% acetonitrile, 0.1% formic acid (FA) and injected into the Dionex UltiMate 3000 HPLC system (Thermo Scientific), equipped with an Acquity UPLC CSH C18 (150 × 2.1 mm, 1.7 μm particle size, 130 Å pore size, Waters, Massachusetts, USA) operated at 40 °C coupled with a Q-Exactive Plus Hybrid Quadrupole-Orbitrap Mass Spectrometer (Thermo Scientific). The Orbitrap was operated in positive mode using data-dependent acquisition. Both MS and MS/MS scan range set was set at 200–1700 m/z, and the inclusion criteria were set to peptides having from two to six charges. A linear elution gradient using solvent A (0.1% FA) and B (0.1% FA in acetonitrile) was applied. The gradient started at 2% B and increased to 45% over 50 min, followed by 2 min at 100% B. The data obtained from the LC–MS/MS was further analyzed by Proteome Discover 3.1 (Thermo Fisher). For database search trypsin digestion was specified, and an in-house library of human milk proteins was used. The error tolerance was set to ± 10 ppm for precursor masses and ± 0.2 Da for fragment ions. Fixed modifications were set to carbamidomethylation on cysteines, and dynamic modifications were set to a maximum of five modifications per peptide, including oxidation of methionine and tryptophan, phosphorylation of serine and threonine.

## Results

### RNA library development

To generate an RNA library of protein-binding RNA molecules that can bind the diverse set of human milk proteins, highly diverse random RNA libraries containing 10^15^ RNA molecules were presented to human milk. This is done through iterative rounds of systematic evolution of ligands by exponential enrichment (SELEX)^24^. Four SELEX RNA libraries were individually enriched against skimmed human milk collected at 3 days, and at 1, 2, and 3 months postpartum, to generate RNA libraries that capture the unique molecular characteristics of each timepoint (Fig. 1). Human milk samples were selected to minimize bias with respect to both sampling time and maternal pre-pregnancy BMI. RNA library sequence diversity was analyzed after four rounds of SELEX using NGS (Supplementary Material, Fig. S1). Round-to-round enrichment analysis revealed that the RNA library obtained after three selection rounds exhibited high sequence diversity suitable for APTASHAPE profiling of human milk, as evidenced by the pronounced dynamic shift observed between rounds 3 and 4 (Supplementary Material, Fig. S1). NGS analysis of the third round libraries yielded 195,822 unique RNA sequences detected at the applied sequencing depth. Importantly, all four SELEX enrichments were initiated from the same well-mixed random RNA library containing 4.5 × 10^16^ RNA molecules, and an amount corresponding to 5 × 10^15^ RNA molecules (8.5 nmol) were extracted 4 times representing a statistically identical sampling of the N_36_ sequence space across time points. The primary variable introduced was the protein composition of the human milk pools (3 days, 1 month, 2 months, and 3 months). Following three rounds of enrichment, the four milk-enriched RNA libraries were mixed equimolarly to generate a combined library for branched selection (APTASHAPE library). This design enables direct comparison between samples while maintaining biological relevance and reproducibility of the selection process.

### Applying APTASHAPE to human milk samples

Branched selection was performed to identify RNA sequences significantly correlated with sample/participant characteristics. This was done by presenting the APTASHAPE library to individual human milk samples. The initial human milk sample cohort, referred to as the ‘discovery cohort,’ consisted of 344 samples from 86 participants, each contributing four samples collected at 3 days, 1 month, 2 months, and 3 months postpartum. The pre-pregnancy BMI of the participants varied from 18.7 to 46.6, which was divided into two groups: 32 with normal BMI (BMI: 18.5–25 kg/m^2^) and 54 with elevated BMI (BMI > 25 kg/m^2^).

The protein-binding RNA molecules were partitioned, subject to cDNA synthesis and prepared for NGS. The NGS analysis identified and quantified the RNA sequences recovered from each sample, with the resulting RNA sequence-binding profiles reflecting protein composition, thereby generating quantitative data on the global protein profile within the discovery cohort. A mean of 3.97 million reads was obtained per sample, and subsequent filtering for sequences likely arising from the same ancestor (Levenshtein–Damerau distance ≤ 3) and RNA sequence representation of ≥ 3 per sample, resulted in a final library of 2429 RNA sequences. These thresholds were implemented to reduce multicollinearity arising from highly similar sequences and to minimize bias due to missing values resulting from limited detection sensitivity. The abundance varied from 25.26 to 0.0007% (Supplementary material Fig. S2). The abundance of the 2429 RNA sequences within each human milk sample was normalized by converting read counts to the percentage of total reads in that sample, allowing for comparison across samples despite differences in sequencing depth. To assess relative variations in RNA sequence binding, each percentage value was further normalized by dividing it by the average percentage value for that RNA sequence across all samples. A resulting value of 1 indicates no deviation from the mean, values below 1 indicate reduced relative binding, and values above 1 indicate increased relative binding. These scaled values were used in all downstream analyses, including OLS, Hierarchical clustering (heatmaps), and principal component analysis (PCA), to facilitate the identification of RNA sequences exhibiting meaningful variation across sample subgroups. The RNA sequences used in this analysis to assess changes in the global protein composition of human milk were strongly enriched in the discovery cohort, providing high confidence in the robustness of their detection (Supplementary Table S1).

Ordinary Least Squares (OLS) linear regression was used to identify RNA sequences significantly correlated with participant characteristics. Statistical significance was determined using a *p* value threshold of 0.05, adjusted for multiple comparisons via the Benjamini–Hochberg false discovery rate (FDR) correction. To account for the anticipated profound changes in protein composition across the sampling times^6,25^, the regression coefficient change threshold for sampling time was set to 1. For maternal BMI-group and parity, both representing more complex heterogeneous conditions with only subtler anticipated subject-specific differences, the regression coefficient change threshold was set to 0.20 to allow for a broader discovery-based search. This resulted in 32, 16, and 85 unique RNA sequences significantly correlated with sampling time, maternal BMI-group, and parity, respectively (Table 1 and Supplementary material, Fig. S3 and Table S2). Notably, for sampling time, a total of 58 sequences were identified across the three OLS comparisons (3 days vs. 1 month, 3 days vs. 2 months, and 3 days vs. 3 months), of which 32 were unique, while the remainder were shared across conditions and exhibited similar relative abundance patterns, which is reasonable since changes in protein composition may reflect milk maturation. Furthermore, all 85 descriptive RNA sequences identified for parity are decreased in primiparous mothers (Table 1).

**Table 1:** Results from the ordinary least squares analysis of the discovery cohort (n = 344), including the 2429 RNA sequences.

Characteristic	Reference/study group	Increased relative abundance of RNA sequence in study group compared to reference	Decreased relative abundance of RNA sequence in study group compared to reference
Sampling time	3 days/1 month	1	11
	3 days/2 months	1	14
	3 days/3 months	13	18
BMI-group	Normal BMI/Elevated BMI	12	4
Parity	Multiparous/Primiparous	0	85

The numbers presented in the table fulfil the thresholds for regression coefficient change of 1 for sampling time and 0.2 for BMI-group and parity, along with a Benjamini–Hochberg adjusted *p* value of 0.05.

### Validation of condition-specific RNA sequence profiles

**Fig. 2:**
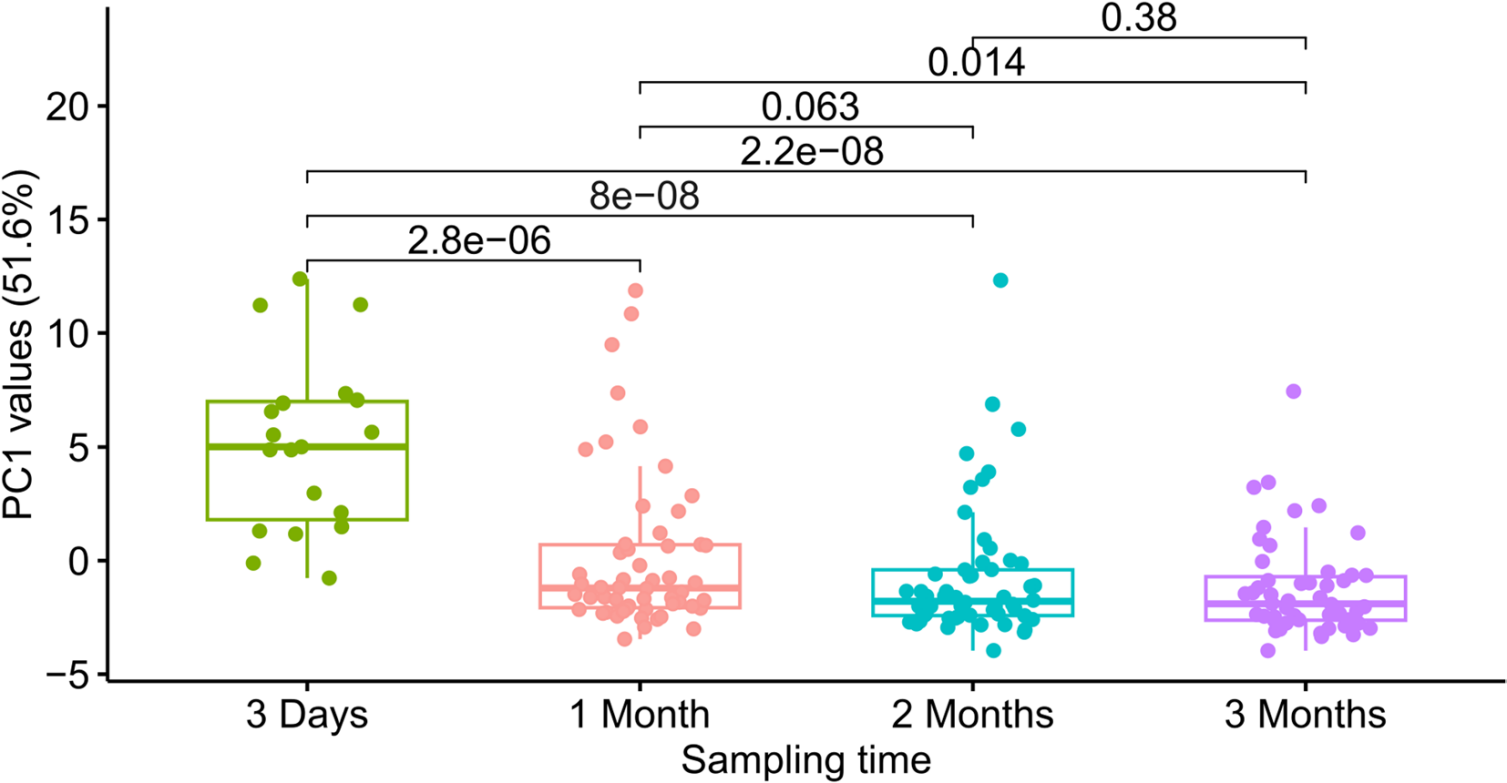
Validation of RNA sequences discriminating sample time. Box plot of PC1 values of samples divided into sampling time extracted from PCA plot for validation of the descriptive RNA sequences identified in the discovery cohort (Supplementary Material, Fig. S4). The difference in median values between 1, 2 and 3 months relative to 3 days was statistically significant, as identified by Wilcoxon rank-sum testing. Furthermore, the difference in median values between 1 and 3 months was statistically significant.

**Fig. 3:**
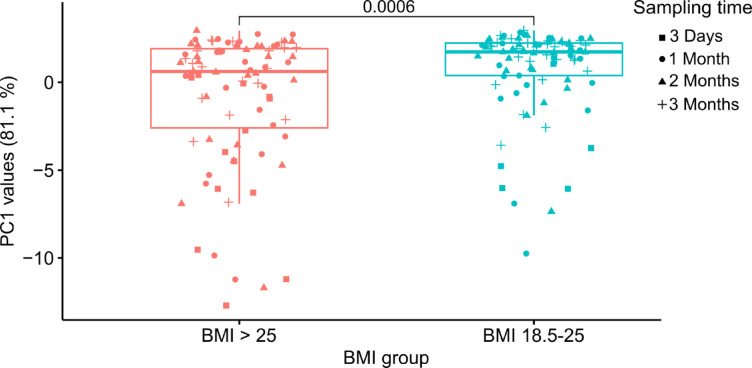
Validation of RNA sequences discriminating normal and elevated BMI status. Box plot of PC1 values of samples divided into elevated BMI (BMI > 25) and normal BMI (BMI: 18.5–25) extracted from PCA plot for validation of the 16 descriptive RNA sequences from the discovery cohort (Supplementary Material, Fig. S5). The difference in median values between normal BMI and elevated BMI was statistically significant (*p* = 0.0006) as identified by Wilcoxon rank-sum testing. Symbols indicate sampling time.

**Fig. 4:**
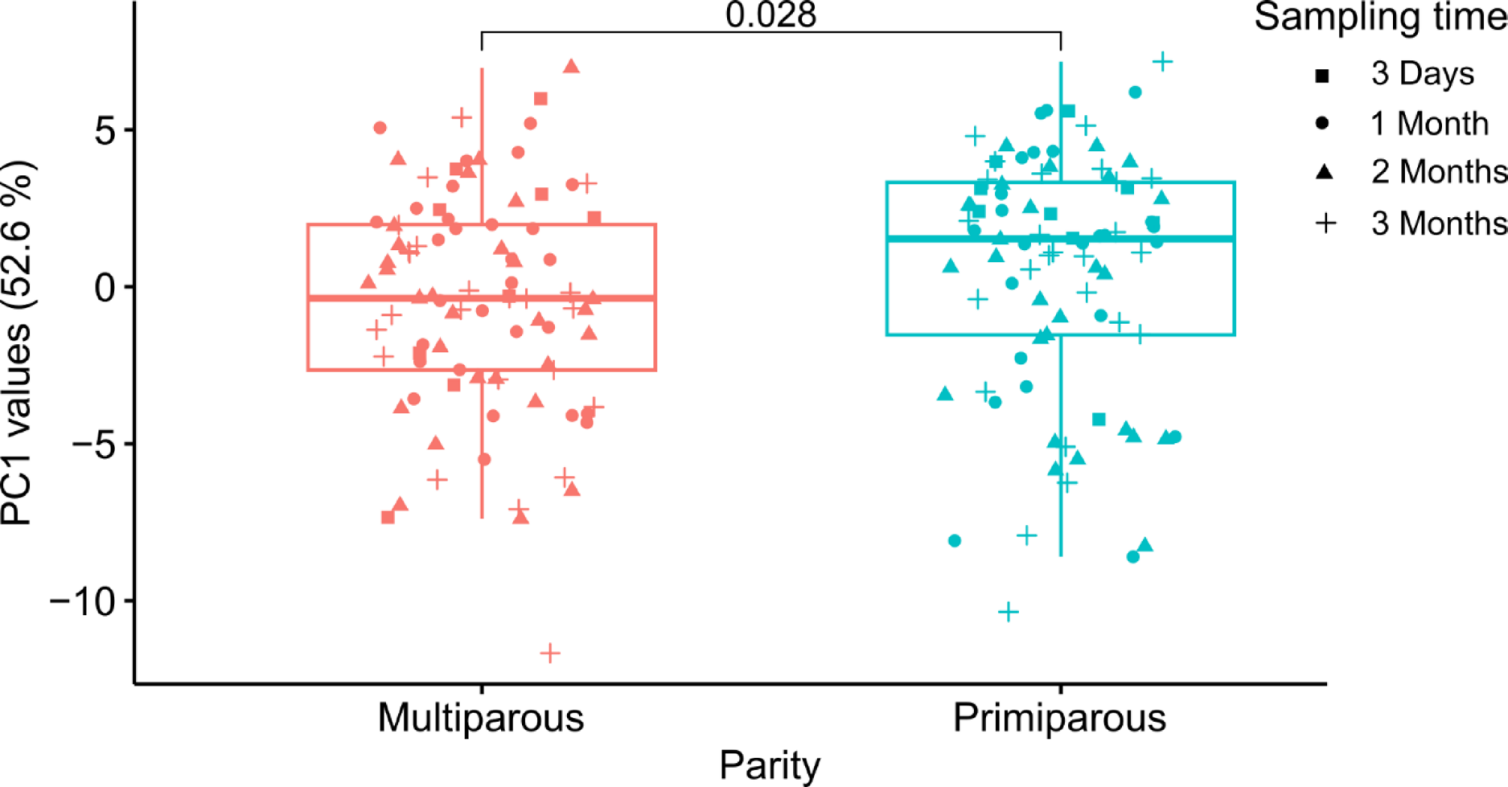
Validation of RNA sequences discriminating parity status. Box plot of PC1 values of samples divided into multiparous and primiparous mothers extracted from PCA plot for validation of the 85 descriptive RNA sequences from the discovery cohort (Supplementary Material, Fig. S6). The difference in median values between primiparous and multiparous was statistically significant (*p* = 0.028) as identified by Wilcoxon rank-sum testing. Symbols indicate sampling time.

To validate the results obtained from the APTASHAPE analysis of the discovery cohort, the APTASHAPE library was applied to a second round of branched selection in a validation cohort also obtained from the MaInHealth cohort. The characteristics of the validation cohort are presented in Table 2. The RNA molecules recovered from the validation samples were analyzed as described for the branched selection on the discovery cohort using NGS (Supplementary Table S3). It was anticipated that the RNA sequences identified as descriptive by OLS in the discovery cohort (Table 1 and Supplementary Table S2) would also be detectable and retain their descriptive value in the validation cohort. Indeed, all descriptive RNA sequences from the discovery cohort were present in the validation cohort. The results of the data analysis showed significant descriptive RNA sequence profiles between subgroups for sampling time, maternal BMI-group, and parity (Figs. 2, 3, and 4). The analysis did not detect any differences in the global protein composition of human milk related to maternal age, C-section, breast infection, maternal secretor status, birth weight, gestational age, infant sex, or infant antibiotic use that could be confirmed in the validation cohort (data not shown).

**Table 2:** Participant characteristics (n = 68) for the validation cohort.

Maternal characteristics	Mean ± Standard deviation	Minimum	Maximum
Maternal age (years)	31.04 ± 3.83	23	41
Maternal height (cm)	169.23 ± 5.51	158	184
Maternal weight (kg)	74.08 ± 14.31	52	118.5
BMI (kg/m^2^)	25.82 ± 4.53	19.33	43.16
Parity (primiparous/multiparous, %)	50.00/50.00		
C-section (%)	16.18		
Breast infection cumulated at 3 months (%)	36.77		
Secretor positive (%)	72.06		
**Infant characteristics**			
Birth weight (kg)	3.73 ± 0.42	2.982	4.844
Gestational age (days)	284.72 ± 7.22	263	296
Infant sex (female %)	50.00		
Infant antibiotics cumulated at 3 months (%)	16.18		
Sample collection (n)			
3 days	18		
1 month	53		
2 months	56		
3 months	49		

Continuous data are represented as means ± standard deviation, and with the minimum and maximum. Categorical data are represented as percentage of total (%) or numbers (n).

#### Sampling time

From the OLS analysis in the discovery cohort 12, 15, and 31 RNA sequences were descriptive when comparing 3 days against 1, 2, and 3 months, respectively. As expected, duplicated sequences occurred, and the analysis identified 32 unique sequences in total. These RNA sequences were similarly found in the validation cohort. A PCA was conducted using the signals of the descriptive RNA sequences in the validation cohort to evaluate their discriminatory power with respect to sampling times (Supplementary Material, Fig. S4). A box plot of the values on the first principal component (PC1) of the PCA for all samples in the validation cohort is shown in Fig. 2. The analysis reveals statistically significant differences between median values of 3 days and 1 month, 2 months, and 3 months, indicating a difference in global protein composition. PC1 separated the 3-day samples from the 1-, 2-, and 3-month groups, while the later time points showed limited separation (Fig. 2). The dispersion of samples along PC1 differed between groups. The 3-day and 1-month samples were more heterogeneous, with within-group variances of 6.57 and 6.78, whereas the 2- and 3-month groups were less heterogeneous, with variances of 4.91 and 4.96. (Fig. 2 and Supplementary Material, Fig. S4).

#### Maternal BMI-group

From the OLS analysis of the discovery cohort, 16 RNA sequences were descriptive of the group compared to the normal weight group (Table 1). These 16 RNA sequences were all present in the validation cohort and were used for a PCA on the RNA sequence levels from the validation cohort (Supplementary Material, Fig. S5). A box plot of the PC1 is shown in Fig. 3, illustrating that the profiles of the 16 descriptive RNA sequences differ significantly between the normal (18.5–25) and elevated (> 25) BMI-group. The box plot also shows that the PC1 values of the elevated BMI-group are more dispersed than the values of the normal BMI-group, with variances of 14.4 and 6.3, respectively.

#### Parity

In addition to sampling time and BMI, the analysis of the RNA sequence levels also showed differences between primiparous and multiparous mothers. The 85 descriptive RNA sequences identified in the discovery cohort were all identified in the validation cohort, where significant differences were also identified between the PC1 values (Fig. 4) obtained from a PCA (Supplementary Material, Fig. S6). No obvious difference in the dispersion of PC1 values is observed for parity groups, with variances of 12.3 and 15.0, respectively.

### Mass spectrometric identification of protein targets bound by descriptive candidate aptamers

To identify protein biomarkers in human milk associated with sampling time, maternal BMI, and parity, affinity purification of human milk proteins was performed using the most abundant candidate aptamer from each RNA sequence family as bait. An RNA sequence family is defined as a group of candidate aptamers sharing a conserved sequence motif, anticipated to bind the same or highly similar protein epitope. Global homology analysis of the significant RNA sequences revealed 16 enriched sequence motifs (Supplementary Table S2), with some showing high similarity. In total, we identified 12 distinct candidate aptamer families anticipated to recognize up to 12 distinct protein epitopes. A subset of 14 candidate aptamers was selected for protein pull-down validation to balance analytical depth and resource feasibility. Selection criteria included: (1) high read counts across both cohorts, (2) conserved sequence motifs within candidate aptamer families (Supplementary Table S2), and (3) representation across at least one of the three descriptive models (sampling time, BMI-group, and parity). The purpose of this targeted validation was to confirm representative aptamer–protein interactions rather than exhaustively identify all targets. Seven of the selected candidate aptamer were associated with parity, two with sampling time, three with BMI, and two linked to both sampling time and BMI. These candidate aptamers were produced as single clones and used for protein pull-down, followed by mass spectrometry to identify their cognate targets and provide biological insights^17,19^.

**Fig. 5:**
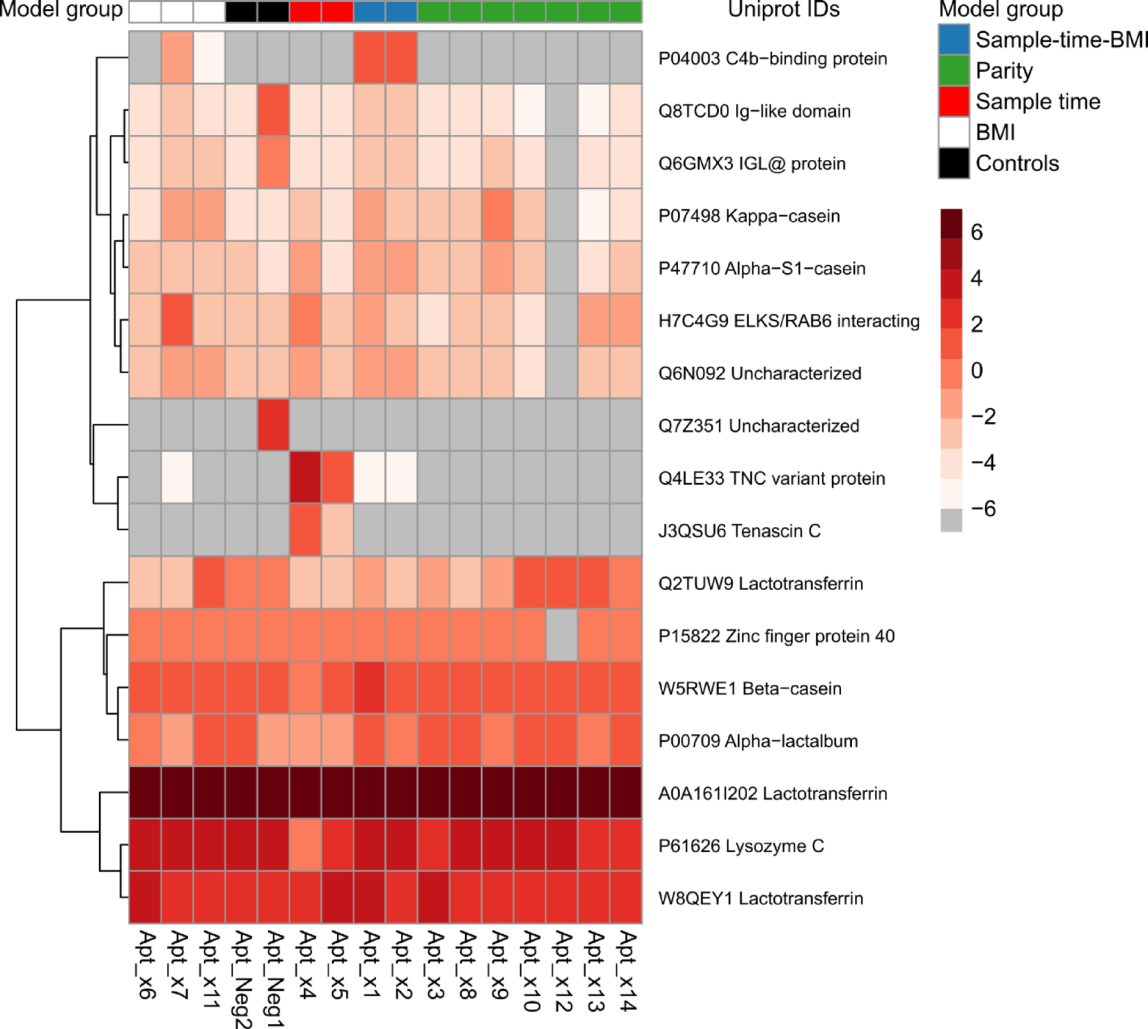
Identification of candidate aptamer protein targets in human milk by LC–MS/MS using 14 most descriptive candidate aptamers for the 4 parameter model groups. Protein pull-downs were performed under native conditions using individual candidate aptamers. Captured proteins were enzymatically digested and analyzed by LC–MS/MS. Data were processed using Proteome Discoverer with searches performed against an in-house human milk protein database. The heatmap displays the log_2_-transformed relative abundance of the 25 most enriched proteins across all candidate aptamer pull-downs, calculated as log_2_(intensity of protein × /total protein intensity per sample × 100). Dark red indicates high enrichment; grey indicates proteins not detected with the respective candidate aptamer. Sample model groups are shown at the top, color-coded accordingly.

The results from the mass spectrometry analysis are presented in a heatmap (Fig. 5). The heatmap shows that the most abundant protein in all pull-downs is lactotransferrin, including the negative controls added to the analysis. The negative control samples, Neg1 and Neg2, were pools of approximately 25 RNA candidate aptamers each, selected against non‑milk targets and not expected to exhibit specificity toward human milk proteins. Similarly, lysozyme C, β-casein, and α-lactalbumin are highly abundant in all pull-downs, suggesting these proteins are nonspecifically retained or co-purified due to very high concentrations in the sample matrix. Interestingly, distinct candidate aptamers from the BMI, sampling time, and sampling time + BMI models yield unique hits for C4b-binding protein and variants of tenascin C (Fig. 5). No unique proteins were detected for the parity model.

## Discussion

Early life nutrition sets the stage for development and later life, warranting for the detailed investigation of human milk protein profiles and their correlation to maternal characteristics. In this study, we present the first application of APTASHAPE, a high-throughput platform exploiting chemically modified RNA sequences ^17,19,20^, to profile the global protein landscape in human milk using samples from 149 participants with more than 500 longitudinally collected samples. In comparison, previous proteomic studies of human milk used MALDI-TOF^16^ or data-independent acquisition mass spectrometry^15,25^, for which sample sizes were substantially smaller, including 53 participants in Viitaharju et al. and 18 participants in both Sánchez-Hernández et al. and Zhang et al.

Sampling time is acknowledged as one of the most important factors for dynamic changes in the human milk composition, supported by multiple studies using different analysis techniques^7,26^. In line with these findings, the current study applied longitudinal sampling, including colostrum at 3 days postpartum and more mature milk samples at 1 month, 2 months and 3 months postpartum and identified sampling time as the strongest driver of variation in the global protein profile. A total of 32 unique RNA sequences showed differences in relative abundance across the sampling times, with a majority showing relatively lower abundance in milk collected at later stages compared to 3 days postpartum (Supplementary Fig. S3 and Table S2). This identified key differentiator between colostrum and mature milk (Fig. 2 and Supplementary Material, Fig. S4) is in line with Sánchez-Hernández et al., which reported larger variability between colostrum and mature milk^16^. Similarly, Zhang et al. identified 2005 proteins in colostrum compared to 1855 in mature milk, with 81 proteins unique to colostrum and 24 unique to mature milk^25^. Additionally, 352 proteins were differentially expressed, with 186 proteins being increased in colostrum^25^. The lower number of RNA sequences with differential binding signal may reflect differences in methodology, population size, and sample handling, but further investigation is required to conduct a direct comparison. Overall, such temporal changes likely reflect physiological adaptations to meet the evolving needs of the growing infant, driven in part by the gradual decline in immunomodulatory proteins over the course of lactation^25,27^.

Next to sampling time, a key focus of this study is the correlation of the milk protein profile to maternal obesity. Sánchez-Hernández et al. reported that, based on two unidentified masses detected by MALDI-TOF, the protein profiles of the overweight/obese group clustered more tightly than those of the normal-weight group^16^. In contrast, the current study observed greater dispersion of the samples’ PC1 values in the elevated BMI group (BMI > 25), based on the signal from descriptive RNA sequences (Fig. 3). Viitaharju et al. investigated differences between overweight and obese mothers and identified 210 proteins that differed between the groups with the majority showing decreased levels in the obese group^15^. In comparison, the present study identified 16 RNA sequences with significant differences between normal BMI (BMI 18.5–25) and elevated BMI (BMI > 25), with the majority being increased in mothers with elevated BMI (Supplementary Material, Fig. S2D). However, Viitaharju et al., did not include normal-weight mothers, which could lead to differences in results. Importantly, the regression coefficient change threshold of 0.2 used here indicates that the detected BMI-related variation is relatively subtle. While this supports an association between maternal BMI and human milk protein composition, the biological implications remain uncertain and should be investigated further.

Additionally, RNA sequence profiles reflecting changes in the human milk protein composition were associated with parity, and distinguished between primiparous and multiparous mothers (Fig. 4). All the significantly different RNA sequences were found in relatively lower abundance in the primiparous mothers compared to the multiparous (Table 1). Previous studies focusing on specific proteins or protein families, such as immunoglobulins and peptide hormones, in human milk have found correlations related to parity. Investigations of the immunoglobulins showed that IgG and IgM showed lower concentrations in multiparous mothers^28^, while lactoferrin concentrations were found to be higher^14^. To our knowledge, no study has consistently reported lower levels of several proteins in primiparous compared to multiparous mothers as observed in the present study, with a relatively lower abundance of the descriptive RNA sequences in primiparous. One hypothesis regarding the relation between parity and human milk composition is that multiparous mothers and infants are affected by the exposures from the other children, which will affect immune components in the milk, but several additional analyses are required to unravel these relationships^29^.

Furthermore, maternal diet was not used as an inclusion or exclusion criterion in the MaInHealth cohort, and although limited dietary data were collected, these were not standardized across all participants and in general showed under reporting in regards to daily calorie intake for adults. A recent systematic review showed that maternal diet does not significantly affect the protein content of human milk^30^. Thus, this relative stability and the large number of participants are expected to minimize the impact of dietary variations in milk protein profiles.

From the pull-down and protein identification analysis, C4b-binding protein and multiple tenascin C isoforms were uniquely detected in the BMI, sampling time, and combined sampling time + BMI models. C4b-binding protein is a part of the complement system and plays a key role in the innate immune system^31^, and was found to be uniquely associated with the BMI and the BMI + sampling time models. This is consistent with the known link between chronic low-grade inflammation and obesity, which often involves upregulation or dysregulation of complement activity^32^. Moreover, since a large proportion of the proteins present in human milk are related to host defense^33^, the association of C4b-binding protein with sampling time is biologically plausible. Tenascin C, also uniquely identified in the sampling time model, is a multifunctional extracellular matrix glycoprotein implicated in several physiological and pathological processes, including tissue repair, wound healing, cardiac injury, and inflammatory responses^34^. In the context of human milk, tenascin C has been shown to neutralize HIV-1, highlighting its role in mucosal immunity and antiviral defense during early infancy^35^. Given the infant’s heightened vulnerability during the neonatal period, the presence of these proteins in colostrum may be essential for supporting protective immune functions. Tenascin was earlier identified in increased levels in colostrum^25^.

In contrast to C4b-binding protein and tenascin C, lactotransferrin, lysozyme C, beta-casein, and α-lactalbumin were consistently identified across all pull-down assays, including negative controls (Fig. 5). The unexpectedly high background binding of these proteins, especially lactotransferrin, suggests a natural affinity for nucleic acids and could be a result of the pull-down strategy used. During APTASHAPE profiling, proteins are initially immobilized on magnetic beads under native conditions, and non-binding proteins are discarded prior to the introduction of the RNA sequence library. However, during the actual pull-down process, candidate aptamers (and not proteins) are immobilized on the magnetic beads. Highly abundant proteins with a natural affinity for RNA can pose a challenge, which is probably the reason for the high background binding of these proteins. Additionally, all pull-down assays are performed under native conditions, meaning that micelles and protein complexes remain intact. Consequently, candidate aptamers may bind not only their intended targets but also associated proteins within complexes, complicating interpretation. The RNA sequence pools used in APTASHAPE profiling of individual samples, were developed against the entire spectrum of human milk proteins under native conditions. Consequently, some candidate aptamers may recognize structural epitopes shared among related proteins or multi-protein complexes, reflecting a “polyclonal” binding behavior rather than strict one-to-one specificity. This behavior is inherent to complex-fluid profiling and aligns with previous applications of APTASHAPE in plasma^17,19^. Therefore, caution is warranted when assigning biological relevance to these interactions, especially for proteins detected in high abundance. Future experiments should aim to orthogonally validate these interactions using recombinant or purified proteins to confirm their specificity and affinities.

Overall, the present study demonstrates the feasibility of APTASHAPE for large-scale profiling of the human milk proteome and highlights both strengths and limitations of the approach. Key strengths include the large number of participants and samples, longitudinal design, and replication of findings in an independent validation cohort, which together increase confidence in the observed associations. Limitations include the modest effect sizes for BMI and parity, potential biases introduced by global protein coupling during APTASHAPE, and challenges in interpreting pull-down data due to complex formation and background binding. Despite these constraints, the results clearly demonstrate the dynamic nature of the milk protein composition, with sampling time exerting the largest influence, followed by subtler associations with maternal BMI and parity. This work establishes a framework for future research linking maternal characteristics, milk composition, and infant health outcomes.

## Supplementary Information

Below is the link to the electronic supplementary material.

Supplementary Material 1

Supplementary Material 2

Supplementary Material 3

Supplementary Material 4

## Data Availability

Data from the study are provided in the Supplementary Information. The datasets generated and/or analysed during the current study are available in the ERDA repository, https://anon.erda.au.dk/cgi-sid/ls.py?share_id=a3W2AgCRwf. Raw sequencing data have been deposited in the European Nucleotide Archive under accession number PRJEB108136.
