# Supplementary material for: Global protein profiling of human milk using pre-enriched RNA-sequence libraries
Source: Sci Rep. 2026 Mar 3;16:11827. doi: 10.1038/s41598-026-41374-w (PMC13066517; doi:10.1038/s41598-026-41374-w)
Supplement: Supplementary file 1 — Supplementary Material 1 [file 41598_2026_41374_MOESM1_ESM.docx]

# Supplementary Figures

Global protein profiling of human milk using pre-enriched RNA-sequence libraries

*Julie Astono^1†^, Asger Givskov Jørgensen^2*†^, Claus Bus^2^, Søren Drud-Heydary Nielsen^3^, Jørgen Kjems^2^, Ulrik Kræmer Sundekilde^1*^*

^1^Department of Food Science, Aarhus University, Agro Food Park 48, Aarhus N, Denmark

^2^Interdisciplinary Nanoscience Center, Department of Molecular Biology and Genetics, Aarhus University, Gustav Wieds Vej 14, Aarhus C, Denmark

^3^Arla Food Ingredients, Sønderupvej 26, Videbæk, Denmark

^*^Corresponding author, ^†^Authors share first authorship


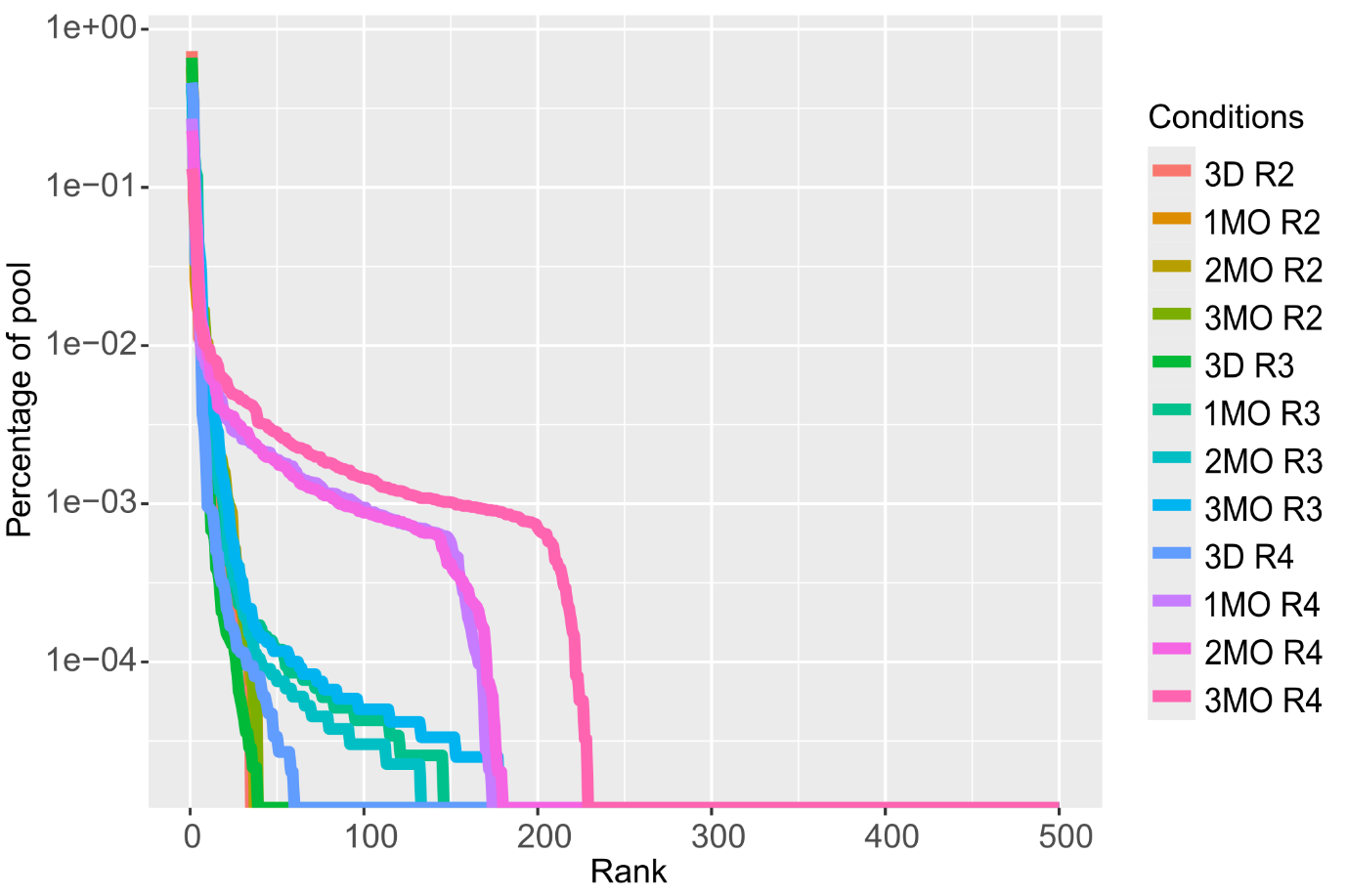


Figure S1. Ranked abundance of RNA sequences in each pool across SELEX rounds 2–4.

This figure shows the ranked abundance of RNA sequences in each pool following SELEX rounds 2 (R2), 3 (R3), and 4 (R4). Within each sample, RNA sequences are ranked individually by abundance, with rank 1 indicating the most abundant sequence, rank 2 the second most abundant, and so on. Abundance is expressed as a percentage of the total reads in each pool. Pools correspond to four postpartum time points: 3D (3 days), 1MO (1 month), 2MO (2 months), and 3MO (3 months).
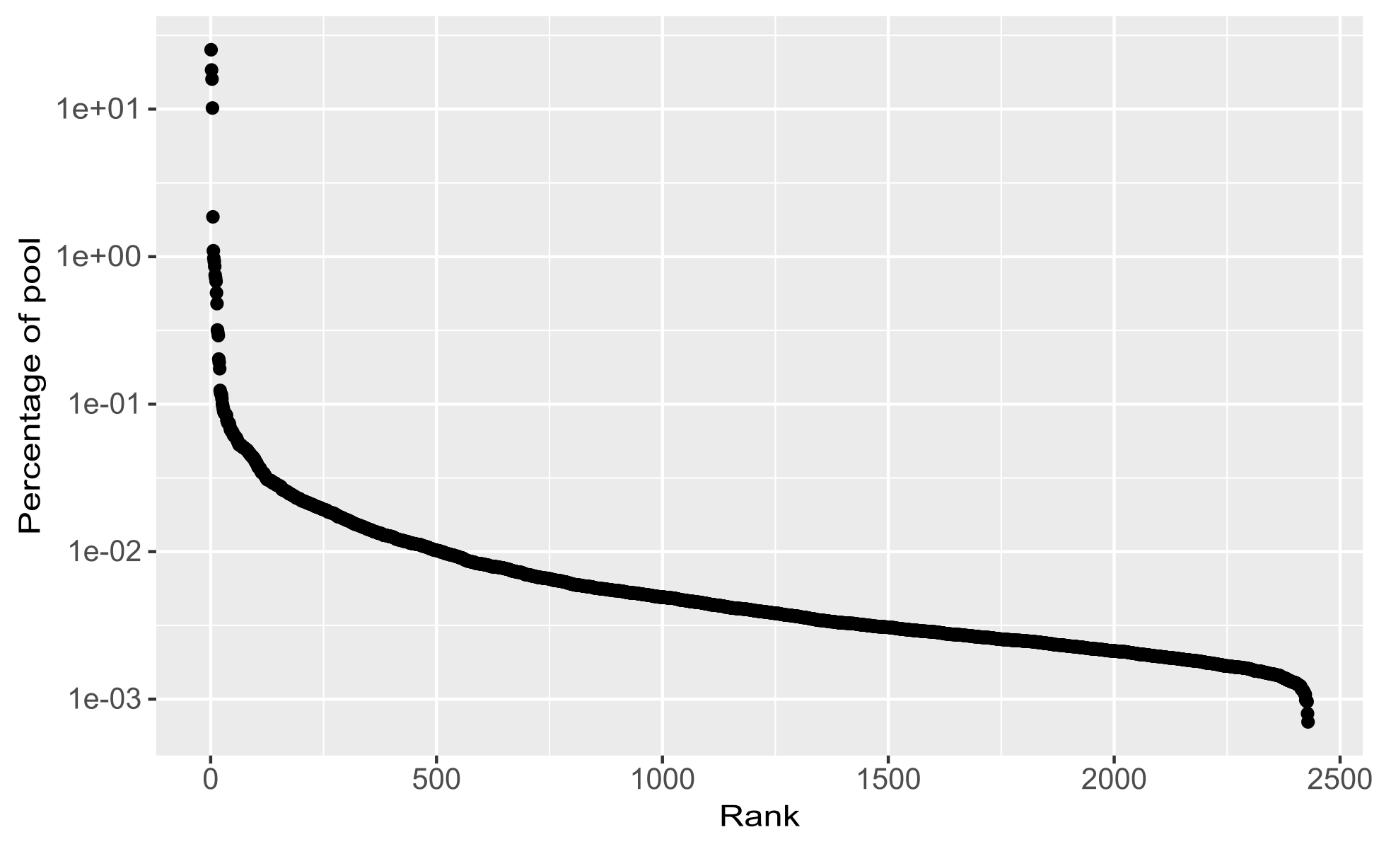


Figure S2. Abundance of RNA sequences following branched selection in the discovery cohort.

The plot displays the abundance of 2,429 RNA sequences after branched selection in the discovery cohort. For each sample, sequence abundance was calculated as the log₁₀ of the percentage, obtained by dividing the total count of each sequence by the total count of all sequences in that sample. RNA sequences are ranked by their total counts across all samples with rank 1 representing the most abundant RNA sequence, and so forth.
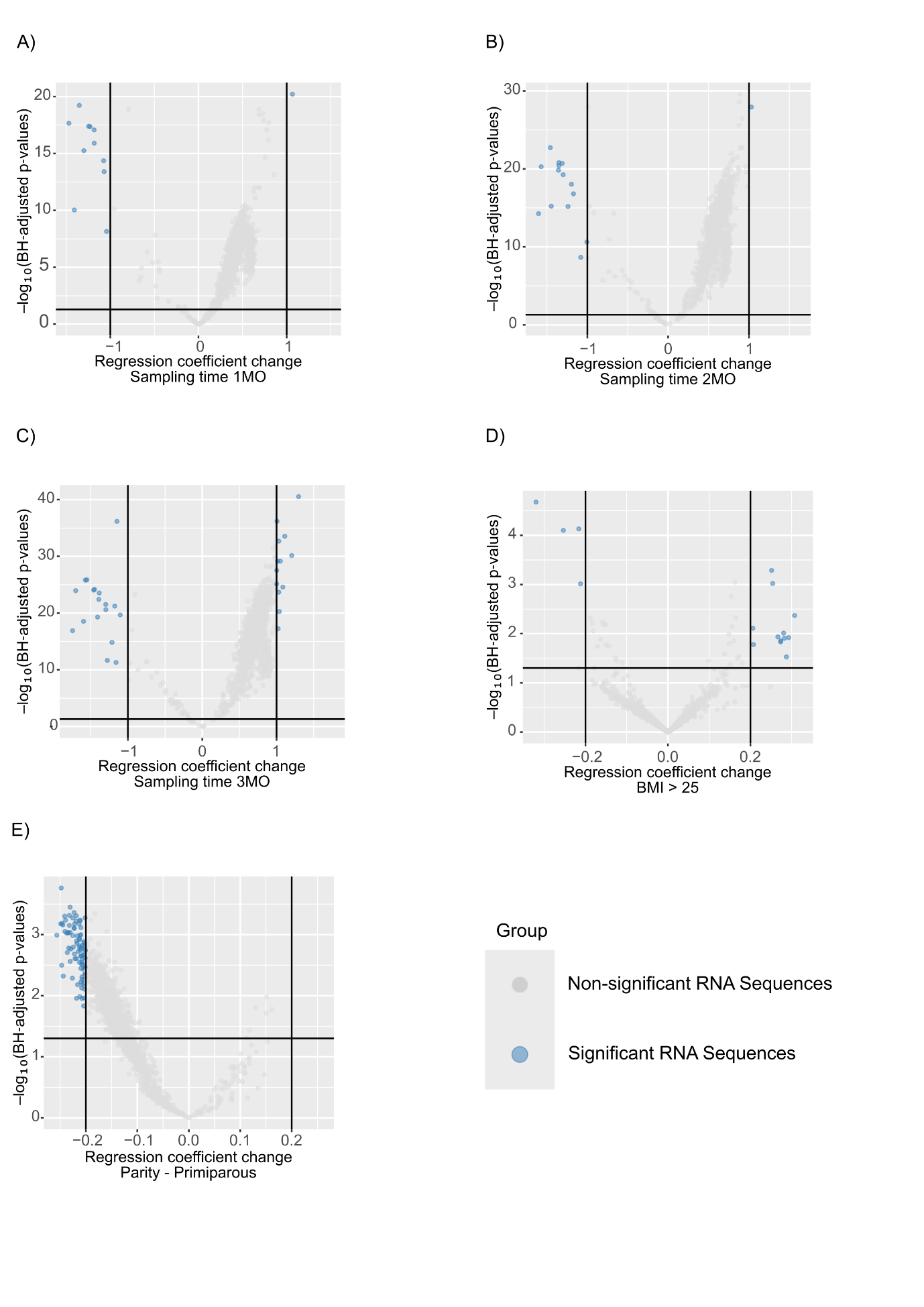


Figure S3: Volcano plots of P-values and regression coefficient changes in OLS analysis across all 344 samples in the discovery cohort using the 2429 RNA sequences. A regression coefficient change (vertical lines) of 1 for sampling time and 0.2 in the BMI-group and parity were applied, along with a Benjamini-Hochberg adjusted P-value of 0.05 (horizontal lines). Statistically significant candidate aptamers are indicated by the blue dots. A) Sampling time: 3 days vs. 1-month samples. B) Sampling time: 3 days vs. 2-months samples. C) Sampling time: 3 days vs. 3-months samples D) BMI-group: normal BMI (18.5-25) vs. elevated BMI (>25). E) Parity: multiparous vs. primiparous mothers.


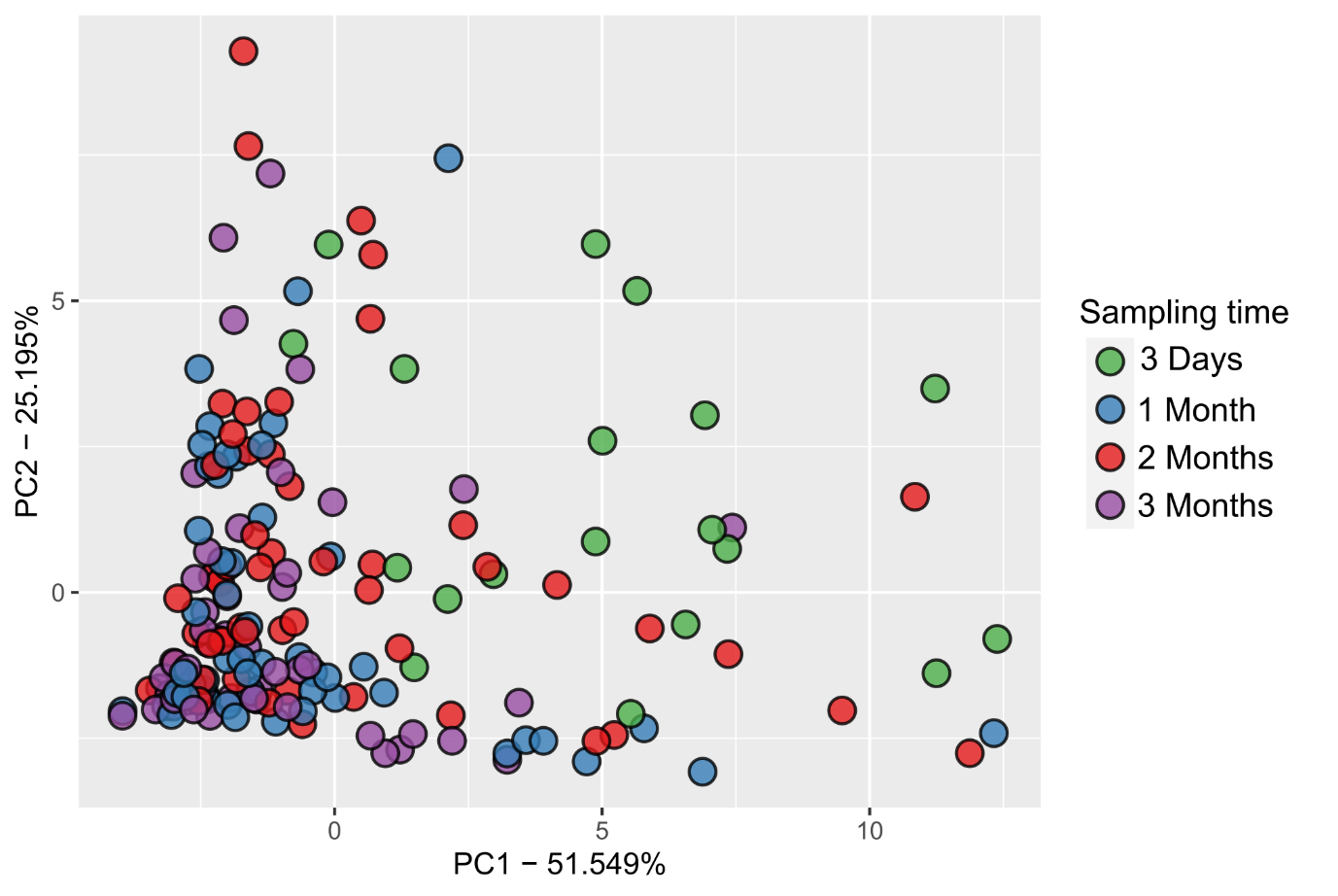


Figure S4. Scores plot of principal components 1 and 2 from the principal component analysis (PCA) of human milk samples from the validation cohort (n=176) based on the 32 descriptive RNA sequences related to sampling time identificed by OLS in the discovery cohort. Colours indicate sampling time: 3D: 3 days (Green), 1MO: 1 month (Blue), 2MO: 2 months (Red), 3MO: 3 months (Purple).


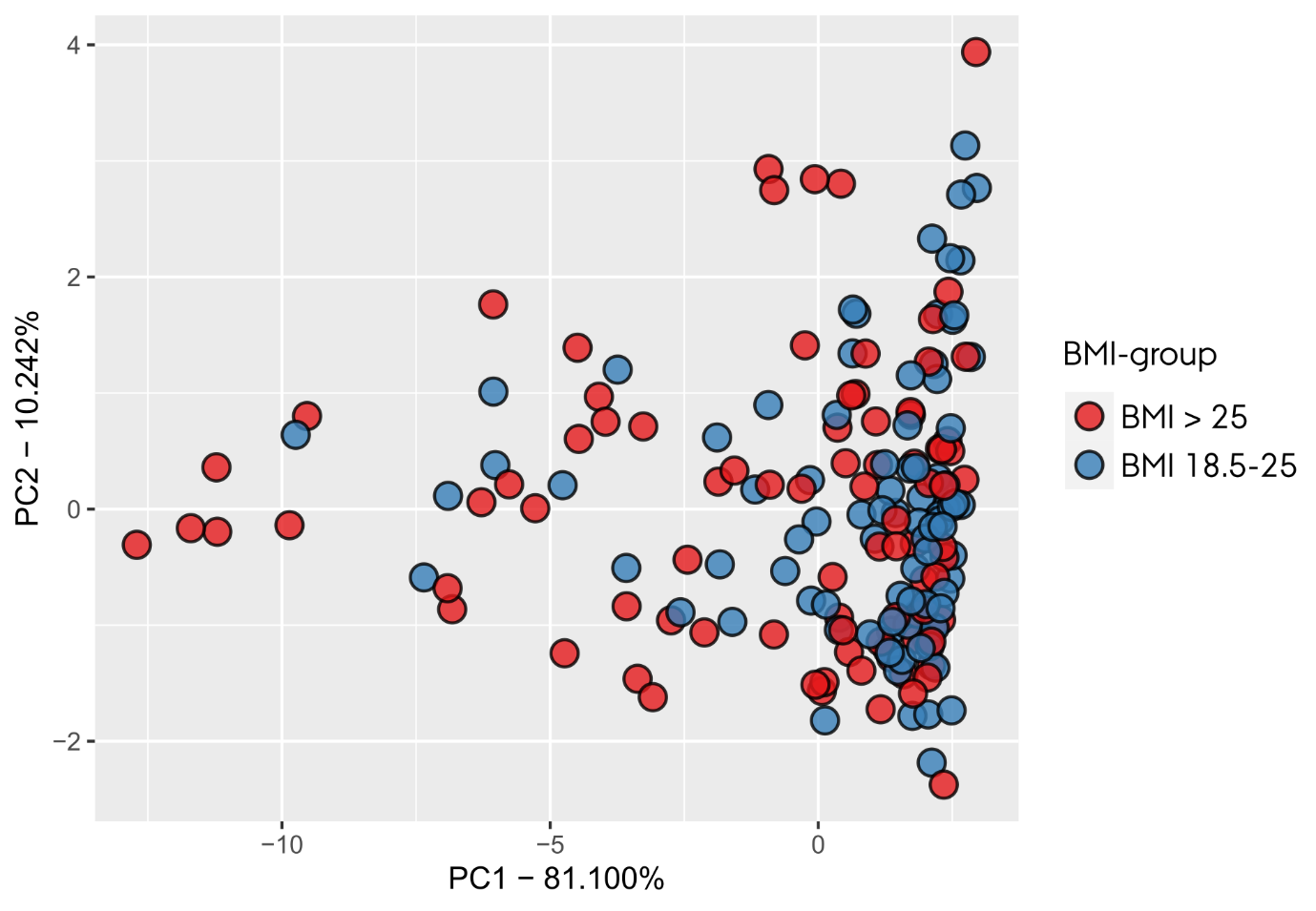

Figure S5. Scores plot of principal components 1 and 2 from the principal component analysis (PCA) of human milk samples from the validation cohort (n=176) based on the 16 descriptive RNA sequences related to BMI-group identified by OLS in the discovery cohort. Blue and Red colours indicate the two BMI-groups.


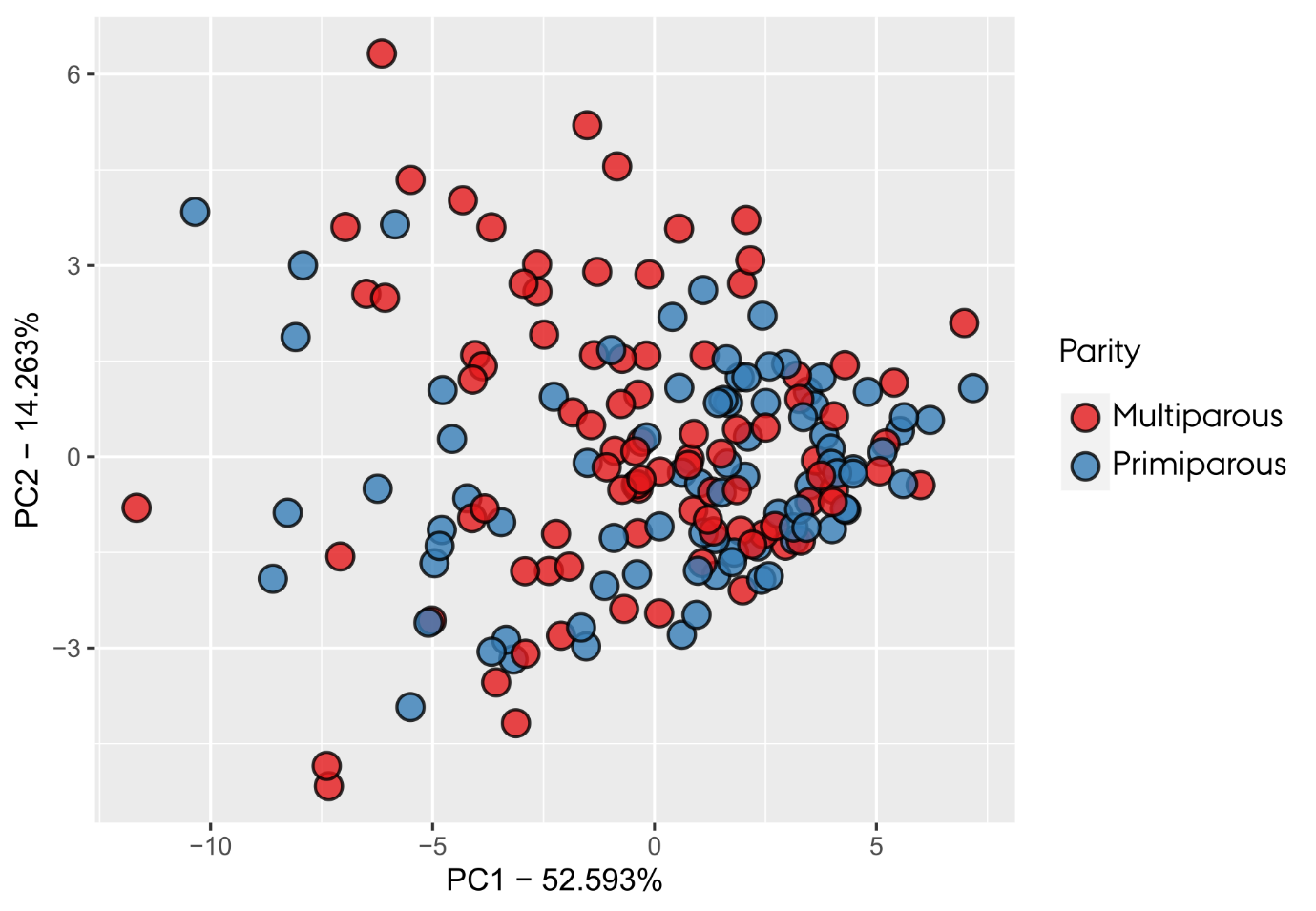


Figure S6. Scores plot of principal components 1 and 2 from the principal component analysis (PCA) of human milk samples from the validation cohort (n=176) based on the 85 descriptive RNA sequences related to parity identified by OLS in the discovery cohort. Blue and Red colours indicate the two parity status.

# Supplemental Tables

**Supplemental Table S1:**External xlsx file.
The RNA sequences from the discovery cohort, after filtering and clustering. These were the RNA sequences used for finding the discriminative RNA sequences using OLS.

**Supplementary Table S2:**External xlsx file.
The change in regression coefficient, negative log10 of p-value, global alignment, de novo motif search results, count number, and duplication matrix of the discriminatory RNA sequences from the OLS analysis shown in Figure S3 and Table 1.

**Supplemental Table S3:**External xlsx file.
The RNA sequences from the validation cohort, after filtering and clustering. These were the RNA sequences used for confirming the capacity of discriminative RNA sequences in an independent experiment.
